# Hypertension, Sarcopenia, and Global Cognitive Function in Community-Dwelling Older Women: A Preliminary Study

**DOI:** 10.1155/2018/9758040

**Published:** 2018-07-02

**Authors:** Hélio José Coelho-Júnior, Bruno Bavaresco Gambassi, Maria-Claudia Irigoyen, Ivan de Oliveira Gonçalves, Paula de Lourdes Lauande Oliveira, Paulo Adriano Schwingel, Cândida Helena Lopes Alves, Ricardo Yukio Asano, Marco Carlos Uchida, Bruno Rodrigues

**Affiliations:** ^1^Applied Kinesiology Laboratory (LCA), School of Physical Education, University of Campinas, Av. Érico Veríssimo, 701 Cidade Universitária “Zeferino Vaz”, Barão Geraldo, 13083-851 Campinas, SP, Brazil; ^2^Hypertension Unit, Heart Institute (InCor), Medical School of University of São Paulo, São Paulo, SP, Brazil; ^3^Center of Health Sciences, University of Mogi das Cruzes, Av. Dr. Cândido Xavier de Almeida Souza, 200 Centro Cívico, 08780-911 Mogi das Cruzes, SP, Brazil; ^4^Physical Education Department, Ceuma University, São Luis, MA, Brazil; ^5^Laboratório de Pesquisas em Desempenho Humano (LAPEDH), Universidade de Pernambuco (UPE), Petrolina, PE, Brazil; ^6^Departamento de Fisioterapia, Universidade Ceuma, São Luis, MA, Brazil

## Abstract

The present study aimed to investigate whether sarcopenia interferes in the association between HTN and cognitive function in community-dwelling older women. One hundred and eleven older women were recruited and dichotomized in hypertensive (*n*=63) and normotensive groups (*n*=48). Volunteers underwent evaluations of the sarcopenic state (i.e., skeletal muscle mass, short physical performance battery (SPPB), balance), hemodynamic parameters, and global cognitive status (i.e., Mini-Mental State Examination (MMSE)). Data demonstrated that hypertensive patients had lower global cognitive status than normotensive subjects. When volunteers were divided according to sarcopenic status, data demonstrated that hypertensive patients with low performance on SPPB (0.006), low values of sarcopenic index (0.03), and low performance on sit-to-stand (0.09) demonstrated poor cognitive status compared with hypertensive patients with normal values of these variables. In conclusion, data of the current study indicate that the sarcopenic state might interfere in the association among hypertension and poor cognitive status, once a higher frequency of hypertensive patients with low lower limb muscle function (i.e., SPPB and sit-to-stand) and muscle mass index (i.e., Janssen index) was observed in the <24 MMSE segment, in comparison with hypertensive patients with normal results in these parameters.

## 1. Introduction

The worldwide projections have indicated an exponential increase in the elderly population in the next 40 years [1]. Indeed, the number of elderly people is expected to reach about 30% of the world population in 2050 [1].

The aging process is a continuous phenomenon accompanied by alterations in some physiological systems (e.g., cardiovascular, skeletal muscle, metabolism), collaborating to the development of geriatric syndromes and chronic diseases. Hypertension (HTN) is one of the most prevalent diseases in older people, affecting more than 70% of this population [2]. The main concern about this disease is its poor prognosis because patients with high blood pressure show an increased risk of stroke (i.e., hemorrhagic and ischemic) and myocardial infarction [2, 3].

However, in the last few years, an increasing number of studies have suggested that HTN is associated not only with elevated cardiovascular risk, but also with cognitive alterations [4–8]. In fact, cross-sectional studies have indicated that hypertensive patients show elevated odds to global and fragmented (e.g., executive function, memory) cognitive impairment than normotensive subjects [4–8]. In this sense, a recent report by the American Heart Association (AHA) shows that a considerable amount of studies have indicated that high blood pressure may promote several changes in the organic system that predispose the genesis of the dementia syndrome, such as Alzheimer's disease (AD) [8]. In addition, the researchers stated that despite recent remarkable progress about this issue, there is still much to know [8].

In turn, similar to HTN, an increased prevalence of sarcopenia—a neuromuscular disease characterized by a progressive muscular atrophy accompanied by low muscle strength and/or lower muscle limb function—is observed in older adults [9, 10]. Sarcopenia is known by its effects on muscular functionality, causing a marked decrease in mobility, transfer capacity, and, consequently, impairing the capacity to perform alone and effectively the activities of daily living [9–11]. Furthermore, besides its close association with impaired muscle functionality, sarcopenic people show elevated risk to present poor cognition [12].

Interestingly, data from population studies have demonstrated that the prevalence of HTN is increased in sarcopenic patients [13]. Moreover, it is worth mentioning that HTN and sarcopenia share several mechanisms present in the genesis of dementia, such as low-grade inflammation and arterial stiffness, which suggest that hypertensive patients with sarcopenia might present lower cognitive scores than nonsarcopenic hypertensive patients [8, 10, 12, 14–16]. However, to the best of our knowledge, no studies have been conducting to test this hypothesis.

Therefore, the present study aimed to investigate whether sarcopenia interferes in the association between HTN and cognitive function in community-dwelling older women.

## 2. Methods

This cross-sectional study was approved by the Research Ethics Committee of the University of Mogi das Cruzes (UMC) under protocol number 621-614 and was developed in accordance with the Declaration of Helsinki and with Resolution 196/96 revised in 2008 of the National Health Council. Since this study aimed at investigating the association between HTN, sarcopenic factors, and cognitive function in community-dwelling older women, volunteers were submitted to morphological, physical, cognitive, hemodynamic, and biochemical evaluations at rest.

### 2.1. Subjects

The study participants were recruited from two specialized healthcare centers for older adults in a town located in the metropolitan area of São Paulo, Brazil. Volunteers were recruited by convenience and asked verbally by the medical team and researchers about their participation in the study. All subjects provided informed consent before enrolment.

The exclusion criteria were use of hormone replacement and/or psychotropic drugs, cerebrovascular disease (e.g., stroke), endocrinal disease (e.g., diabetes), pulmonary disease, neurological or psychiatric disease (e.g., Parkinson's or Alzheimer's disease), musculoskeletal disorders, comorbidities associated with greater risk of falls, and any kind of dizziness, blurred vision, or lightheadedness when rise or remain standing for long, which could indicate orthostatic hypotension and/or labyrinthitis. The inclusion criteria were age ≥ 60 years, live in the community, 4–7 years of schooling, and be independent to perform the instrumental (IADL) and basic (ADL) activities of daily living. After the application of the exclusion and inclusion criteria, 111 older women were included in the analyses.

The volunteers were subdivided into normotensive and hypertensive groups according to the previous clinical diagnosis of HTN. Since both healthcare centers serve a large number of patients, and the medical team (i.e., nurse, physician and physical educator) is of limited size, the pathological conditions were simply recorded by the head physician and head nurse of each center. A specialist (i.e., cardiologist) who was not affiliated to and was outside the center then made the diagnosis of HTN, according to the guidelines [17].

## 3. Morphological, Physical, Hemodynamic, and Biochemical Evaluations

All subjects were instructed to refrain from physical exercise for 96 hours before the tests and to refrain from eating or drinking (including water) for 8 hours before the tests. All tests were conducted between 07:00 am and 10:00 am under a controlled temperature of 24°–26°C [18].

### 3.1. Assessment of Body Composition

A data acquisition system (Tanita InnerScan 50v, Tokyo, Japan) was used to measure the bioelectrical impedance. This system uses an electrical current to quantify the amount of intracellular and extracellular water in the body. The device has four electrodes: two are placed on the feet while the other two are placed in the hands of the volunteers. The device measures body mass index (BMI), total body mass, total muscle mass, and appendicular muscle mass. Janssen and Baumgartner muscle mass indexes were evaluated according to the following equations: Janssen index = absolute muscle mass/height^2^; Baumgartner index = appendicular muscle mass/height^2^ [10, 11, 18].

### 3.2. Short Physical Performance Battery

The SPPB includes three tests that evaluate lower-body function: a hierarchical test of standing balance, a 4-meter walk speed test, and a five repetitive chair stands. Each SPPB component test (balance, gait, and chair stand) is scored from 0 to 4 with a score of 0 representing the inability to perform the test and a score of 4 representing the highest category of performance. For the balance tasks, the participants were asked to stand with their feet side by side, followed by the semitandem (heel of one foot alongside the big toe of the other foot) and tandem (heel of one foot directly in front of and touching the other foot) positions for 10 s each. For gait speed, a 4-meter walk speed test at the participant's usual pace was timed. For the ability to rise from a chair, volunteers were requested to rise from a chair five times as fast as possible with their arms crossed in front of the body. The stopwatch was activated when the volunteer raised their buttocks off the chair and was stopped when the volunteer seated back. A summary performance score was obtained by adding the scores of each individual SPPB component test (range 0–12), with higher scores indicating better lower-body function [19–21].

### 3.3. Evaluation of Hemodynamic Parameters at Rest

The procedures for measurement of blood pressure were adapted from the VII Joint National Committee on Prevention, Detection, Evaluation, and Treatment of High Blood Pressure (JNC7) [22]. In summary, older women remained in a sitting position on a comfortable chair for 15 minutes in a quiet room. After this period, an appropriate cuff was placed at approximately the midpoint of the upper left arm (heart level). An automatic, noninvasive, and validated [23] arterial blood pressure monitor (Microlife-BP 3BT0A, Microlife, Widnau, Switzerland) was used to measure systolic blood pressure (SBP), diastolic blood pressure (DBP), and heart rate (HR). During blood pressure recording, volunteers remained relaxed in the sitting position, with parallel feet at one shoulder width, both forearm and hands on the table, supinated hands, back against the chair, without move or talk. The volunteer did not have access to blood pressure values during measurement. The evaluation lasted approximately 80 seconds and was performed three times with one minute of rest among the measurements. The mean of measurements of each volunteer was used in the final analysis. Mean arterial pressure (MAP), double product (DP), and pulse pressure (PP) were evaluated according to the following equations: MAP = [SBP + (2 × DBP)]/3, DP = SBP × HR, and PP = SBP – DBP. The size of the arm cuff was selected after measuring the arm circumference of each participant (Sanny, São Paulo, Brazil).

### 3.4. Saliva Collection

Saliva samples were collected at rest using a roller cotton (Cremer, São Paulo, Brazil). Researchers asked the participants to put a piece of cotton in their mouth and remove it when it became soggy. The samples were transferred to a Falcon tube and frozen immediately until the end of the experiment. In the laboratory, the samples were centrifuged and supernatants were stored at −80°C for posterior analyses using the Griess colorimetric method [24].

### 3.5. Measurement of Nitric Oxide (NO)

The NO pool was considered the mensuration of nitrite (NO_2_^−^) levels in saliva. Briefly, a solution containing 0.1% N-(1-naphthyl)-ethylenediamine (NED) (Sigma) and 1.0% sulfanilamide (Sigma) was prepared in 2.5% phosphoric acid as the diluent. Saliva samples (50 *μ*L) and Griess reagent (50 *μ*L) were mixed and transferred to microplates. Absorbance was measured at 530 nm, and sodium nitrite (NaNO_2_) was used as the standard. Nitrite quantification (*µ*M) was achieved using a standard curve constructed with NaNO_2_ at the concentrations of 100, 50, 25, 12.5, 6.25, 3.13, 1.66, and 0 *µ*M [24]. Data were analysed using the Microplate software (CA, USA).

## 4. Questionnaires

All interviews were performed face to face, so that the application of the tests, along with their objectives, was explained individually to each participant. If a question was not understood, the interviewer repeated the question more slowly, up to three times.

### 4.1. Mini-Mental State Examination (MMSE)

The participants' cognitive function was assessed using the MMSE, which is a standard test in cognitive aging research to assess mental status with a possible score of 0–30 [16]. MMSE evaluates orientation, registration, and short-term recall, attention and concentration, language (naming, sentence writing, and comprehension), and visuospatial abilities. Individual items are summed to generate the total score. If individuals decline or are unable to attempt a task, the value of that particular item would be missing. The MMSE was applied according to Brucki et al. [25].

### 4.2. Geriatric Depressive Scale (GDS)

The quantification of depressive symptoms was evaluated by the short version of the geriatric depression scale (GDS). The scale comprises 15 questions about the feelings, or the frequency of the feelings, of the patient in front of some conditions of the life. The answers are based on a binary code: yes or no [26, 27].

### 4.3. Instrumental (IADL) and Basic (ADL) Activities of Daily Living

ADL and IADL were evaluated through Katz and Pfeffer index, respectively. The Katz ADL measures six self-care tasks using a dichotomous rating (dependent = 0; independent = 1) in the hierarchical order of decreasing difficulty as listed: bathing, dressing, toileting, transferring to and from a chair, maintaining continence, and feeding. A final score of six indicates *independency*, whereas a 0 score indicates a full dependence [28]. The Pfeffer questionnaire constitutes of a 10-item instrument. Each item is scored on a scale of 0 (independence) to 3 (dependence), where higher scores reflect greater dependency of the patient [29].

## 5. Statistical Analysis

To determine the differences in the continuous data between the groups (i.e., normotensive and hypertensive), Student's *t*-test for independent samples was performed. Comparisons between the groups according to the variables associated with sarcopenia were made using one-way analysis of variance (ANOVA) followed by Dunnett's post hoc test. A posteriori, chi-square test was performed to investigate the association between the dependent categorical variable (i.e., MMSE) and the independent categorical variables. The median values were chosen as the cutoff values. Multiple linear regression was applied to examine how the variability in MMSE could be explained by sarcopenic state and HTN. To be considered as an independent variable associated with HTN, the results were required to have a *P* ≤ 0.05. All analyses were conducted using the IBM SPSS Statistics, version 20.0, software (IBM Corp., Armonk, NY, USA).

## 6. Results

Table 1 shows the characteristics of older women according to HTN. As expected, volunteers for both groups were elderly (>60 years old). BMI evaluation indicated that both groups showed an overweight phenotype. Regarding sarcopenia, groups demonstrated normal skeletal muscle indexes (i.e., Janssen and Baumgartner), as well as intermediate performance on SPPB [10]. However, walking speed values suggested a poor mobility in the hypertensive group (≤0.8 m/s), whereas normal results were found in the normotensive group (>0.8 m/s). Katz and Pfeffer's analyses indicated that volunteers for both groups showed an elevated level of independence to perform the ADL and IADL. Depressive symptoms were in an acceptable range of normality according to GDS. Lastly, suspected levels of dementia were found in the hypertensive group (<24 points) [30]. After application of Student's *t*-test, no significant differences were observed between normotensive and hypertensive older women regarding the anthropometric parameters and physical functional tests. However, the hypertensive group showed increased SBP, MAP, and DP, as well as reduced scores on the MMSE and salivary bioavailability of NO in comparison with normotensive volunteers.

Table 2 shows the dichotomized (i.e., *high* and *low*) results on MMSE according to the variables associated with sarcopenia. In line with the unadjusted analysis (i.e., Table 1), hypertensive patients showed lower results in MMSE, regardless of anthropometric characteristics, physical function tests, and sarcopenia. Moreover, analysis of *sarcopenic* domain indicated a possible association among hypertension, appendicular skeletal mass plus SPPB, and low scores in the MMSE.

The associations between the categorical variables using the chi-square test (*χ* ^2^) are shown in Table 3. Data demonstrated that hypertension plus low performance on SPPB (0.006), HTN (0.012), HTN plus low values on Janssen index (0.03), and HTN plus low performance on sit-to-stand (0.09) (tendency) were significantly associated with MMSE.

Table 4 shows the results from multiple linear regression to predict MMSE. Results indicate that MMSE variability was not explained by morphological (i.e., absolute muscle mass, appendicular skeletal muscle mass, Janssen index, and Baumgartner index) or functional (i.e., walking speed, SPPB, and sit-to-stand) parameters. On the other hand, variability in TUG was significantly explained by HTN (18%).

## 7. Discussion

In the current study, hypertensive patients scored lower in the MMSE than normotensive patients. In addition, our findings indicate that sarcopenic state can interfere in this phenomenon, given that a higher frequency of hypertensive patients with reduced lower limb muscle function (i.e., SPPB and sit-to-stand (tendency)) and muscle mass index (i.e., Janssen index) was observed in the <24 MMSE segment when compared to hypertensive patients with normal results in these parameters.

Several experiments are in line with the findings of the present study and have demonstrated a negative association between HTN status and high blood pressure values with poor cognitive performance in older adults [4–7]. In the experiment of Obisesan [6], for example, the authors investigated the association between HTN status and cognitive performance using data from the Third National Health and Nutrition Examination Survey (NHANES III). As in the present study, results demonstrated that hypertensive patients showed a lower global cognitive score (i.e., MMSE) than normotensive patients. However, an increasing number of studies have extended these results and demonstrating that HTN is associated with a low functioning of numerous cognitive domains, such as executive function, praxis, learning, and memory [5, 7].

Unfortunately, we were unable to conduct cognitive-specific analysis to indicate what cognitive domains were associated with HTN and sarcopenia. It is noteworthy that MMSE is a global cognitive test, which does not contemplate the evaluation of some crucial cognitive domains to successful aging, such as executive function [8]. However, to the best of our knowledge, this is the first study aimed at investigating the association between HTN, sarcopenia, and cognitive performance, and future studies should be based on these seminal findings, along with the aforementioned data, and explore specific cognitive domains that demonstrate association with HTN (e.g., executive function).

In relation to sarcopenia, multiple linear regression results did not indicate a significant effect of sarcopenic parameters on MMSE variability. On the other hand, chi-square test showed that low lower limb muscle function (i.e., SPPB and sit-to-stand) and muscle mass index (i.e., Janssen index) might interfere in the relation between HTN and cognitive function. These results are contradictory and should be interpreted with caution. Indeed, our sample is of limited size to perform a multiple linear regression analysis and our findings may be being underestimated, while a simple frequency of distribution of sarcopenic parameters, such as chi-square test, seems to be a better approach in this context.

Contrary to HTN, the association between sarcopenia and cognitive dysfunction has just recently begun to be demystified. In fact, only findings from 7 cross-sectional studies were analysed in a systematic review with meta-analytic regression, which demonstrated that the odds of poor global cognitive scores (i.e., MMSE) in sarcopenic patients were 2.6-fold higher than in non-sarcopenic patients [12]. In addition, longitudinal studies have demonstrated that sarcopenic patients showed an increased risk for the development of dementia in short-term follow-up periods (i.e., 1 year) [16, 31].

It is noteworthy that, in the study of Moon [31], low SPPB scores, but not low muscle mass, was associated with high risk of developing dementia. In the current study, volunteers with HTN and low performance on SPPB demonstrated a lower *P* value (0.0006) than those with HTN and low Janssen index (0.03) in the <24 MMSE segment, suggesting that lower limb muscle function might be more important than skeletal muscle mass in the context of HTN and cognitive function. Findings from Lee [32] support this hypothesis, given that low scores in physical tests were associated with cognitive impairment and atherosclerotic markers, while no significant associations were observed with low muscle mass.

Moreover, these findings indicate a possible biological process underlying the association between HTN, low lower limb muscle function, and poor cognition observed in the present study because atherosclerosis is one of the leading causes of dementia due to its strong association with vascular remodelling and stiffening [8, 33]. Interestingly, several studies performed by our group [11, 34] and other groups [14, 15, 35] have demonstrated that low muscle mass—combined or not with decreased muscle function—is associated with indirect measurements of arterial stiffness in older adults (e.g., pulse pressure), suggesting that the influence of the sarcopenic factors in the relationship between HTN and cognitive status is mediated by similar mechanisms. This hypothesis was recently confirmed in the elegant study performed by Kohara et al. [35], which observed that low muscle mass was strongly associated with poor cognitive scores and arterial stiffness.

In an attempt to understand whether these aforementioned mechanisms might be responsible for the results observed in the current study, we performed a new series of analyses (Supplementary Figure 1) evaluating PP levels and salivary NO bioavailability according to SPPB, sit-to-stand, and SMI in the hypertensive volunteers. Our findings indicate that hypertensive patients with impaired performance in physical tests (i.e., SPPB and sit-to-stand) showed lower NO bioavailability than hypertensive patients with normal performance, but just sit-to-stand reached significant results (*P* < 0.05). On the other hand, *P* values were quite similar among the groups. Therefore, these findings indicate a possible mediation of NO in the results observed in the present study.

In fact, NO is an important vasoactive substance synthesized and released by the vascular endothelium affecting arterial morphology and functioning under physiological and physiopathological conditions [36–38]. However, its acute and chronic pharmacological (e.g., L-NAME, L-NMMA) or physiological inhibitions in humans and animals cause severe alterations in the vascular homeostasis, as, for example, reduction in the vascular cross-sectional area, compliance, as well as increase in pulse-wave velocity—a well-established marker of arterial stiffness [36–38]. Furthermore, numerous studies have argued a possible role of NO in the pathogenesis of dementia syndromes (e.g., Alzheimer's disease) through neurotoxicity and neuroprotective mechanisms [39].

Some limitations of the present study should be mentioned and addressed in future studies to a better understanding of the relation between HTN, sarcopenia, and cognitive function in community-dwelling older women, including our limited sample size and a number of cognitive evaluations, which avoid us to perform more robust statistical analysis, like logistic and multiple regressions. In addition, NO was the only possible mechanism investigated in the present study and several risk factors for the development of cognitive dysfunction are shared by HTN and sarcopenia, all of which may be responsible for the seminal results showed in the current study, such as inflammation, oxidative stress, and sedentary lifestyle.

## Figures and Tables

**Table 1:** Characteristics of older women according to hypertension.

Variables	Total (*n*=111)	
	Normotensive	Hypertensive
	(*n*=48)	(*n*=63)
*Anthropometric characteristics*		
Age (years)	65.5 ± 2.0	65.1 ± 1.9
Weight (kg)	63.8 ± 10.9	66.4 ± 10.4
BMI (kg/m^2^)	26.0 ± 4.1	27.5 ± 4.5
Body fat (%)	32.7 ± 9.5	34.4 ± 7.3
Absolute muscle mass (kg)	41.4 ± 4.5	39.9 ± 7.6
Appendicular skeletal muscle mass (kg)	17.7 ± 4.6	17.4 ± 5.6
Janssen index (kg/m^2^)	65.7 ± 6.5	63.0 ± 7.0
Baumgartner index (kg/m^2^)	11.7 ± 1.6	11.9 ± 2.0

*Physical functional tests*		
Sit-to-stand (s)	15.2 ± 3.3	15.3 ± 3.3
Walking speed (m/s)	0.84 ± 0.33	0.72 ± 0.34
Side-by-side stand	9.7 ± 1.1	9.7 ± 1.1
Semitandem stand	9.7 ± 1.2	9.6 ± 1.4
Tandem stand	9.4 ± 1.6	8.7 ± 2.6
Short physical performance battery	9.6 ± 1.2	9.3 ± 1.5

*Hemodynamic parameters*		
SBP (mmHg)	126.0 ± 11.4	150.3 ± 18.0^*∗*^
DBP (mmHg)	77.1 ± 9.0	87.5 ± 10.1
MAP (mmHg)	93.4 ± 8.3	106.6 ± 17.6^*∗*^
HR (bpm)	75.3 ± 10.0	76.8 ± 11.5
DP (mmHg·bpm)	9508 ± 1561	11373 ± 2683^*∗*^
PP (mmHg)	49.9 ± 11.7	62.4 ± 16.6^*∗*^

*Clinical parameters*		
ADL	0.15 ± 0.36	0.10 ± 0.30
IADL	2.0 ± 2.1	2.8 ± 3.8
GDS	4.9 ± 6.4	4.0 ± 4.6
MMSE	25 ± 2	23 ± 3^*∗*^
Average of drugs	0.31 ± 0.62	0.59 ± 0.79

*Biochemical parameters*		
Salivary NO (*µ*M)	150.3 ± 97.0	110.5 ± 67.9

BMI = body mass index; SBP = systolic blood pressure; DBP = diastolic blood pressure; MAP = mean arterial pressure; HR = heart rate; DP = double product; PP = pulse pressure; ADL = activities of daily living; IADL = instrumental activities of daily living; GDS = geriatric depressive scale; MMSE = Mini-Mental State Examination; NO = nitric oxide. ^*∗*^ *P* < 0.05.

**Table 2:** MMSE according to the presence of hypertension and high or low levels of variables related to sarcopenia.

	Total (*n*=111)			
	Normotensive		Hypertensive	
Variables	High MMSE	Low MMSE	High MMSE	Low MMSE

*Anthropometric characteristics*				
Absolute muscle mass (kg)	25.7 ± 2.1	26.3 ± 2.7	21.8 ± 5.6	20.1 ± 8.8^a^
Appendicular skeletal muscle mass (kg)	25.6 ± 2.0	25.5 ± 3.5	20.3 ± 1.7^a^	23.3 ± 7.5
Janssen index (kg/m^2^)	26.4 ± 2.4	26.0 ± 1.7	23.0 ± 3.8^a^	22.7 ± 2.8^a^
Baumgartner index (kg/m^2^)	25.8 ± 2.4	25.2 ± 2.8	23.0 ± 3.2^a^	24.0 ± 3.4

*Physical functional tests*				
Sit-to-stand (s)	25 ± 2.9	26.1 ± 2.2	21.2 ± 7.1^a^	22.3 ± 6.8
Walking speed (m/s)	25 ± 2.6	25.0 ± 2.6	21.6 ± 6.7	21.7 ± 7.9
Short physical performance battery (points)	24.6 ± 3.6	25.7 ± 2.4	24.8 ± 2.9	22.9 ± 3.2^b^

*Sarcopenia*				
Absolute muscle mass plus walking speed	24.8 ± 2.3	28.0 ± 0.0	22.8 ± 2.4^b^	23.6 ± 0.5
Absolute muscle mass plus sit-to-stand	25.1 ± 1.7	27.1 ± 2.7	22.4 ± 3.5^b^	24.2 ± 3.5
Absolute muscle mass plus short physical performance battery	24.3 ± 3.2	24.2 ± 1.2	26.3 ± 2.7	—
Appendicular skeletal muscle mass plus walking speed	24.8 ± 2.3	24.3 ± 3.5	22.3 ± 2.3	25.3 ± 2.5
Appendicular skeletal muscle mass plus sit-to-stand	25.4 ± 1.8	27.0 ± 2.7	22.2 ± 3.3^ab^	24.3 ± 3.9
Appendicular skeletal mass plus short physical performance battery	25.0 ± 3.5	24.2 ± 1.2	26.0 ± 3.0	19.0 ± 0.0^ac^
Baumgartner index plus walking speed	24.7 ± 2.9	28.0 ± 00	23.1 ± 3.3	24.6 ± 3.7
Baumgartner index plus sit-to-stand	26.3 ± 2.6	27.0 ± 2.9	23.1 ± 2.9^a^	23.6 ± 2.8
Baumgartner index plus short physical performance battery	24.6 ± 3.3	24.2 ± 1.2	25.7 ± 2.4	25.0 ± 3.9
Janssen index plus walking speed	25.0 ± 2.7	26.3 ± 0.5	22.5 ± 2.2^b^	23.5 ± 1.4
Janssen index plus sit-to-stand	25.1 ± 1.7	25.2 ± 1.6	22.5 ± 3.5	21.8 ± 4.0
Janssen index plus short physical performance battery	23.4 ± 3.1	28.0 ± 0.0^a^	25.7 ± 1.5	24.0 ± 0.0

^a^ *P* < 0.05 versus high NTS; ^b^ *P* < 0.05 versus low NTS; ^c^ *P* < 0.05 versus high HTS; ^d^ *P* < 0.05 versus low HTS; data are shown as mean ± SD; MMSE = Mini-Mental State Examination.

**Table 3:** Frequency (%) of the distribution of older women according to MMSE.

Variable	MMSE		*P*
	<24	≥24	
Hypertension			
Yes	24.4	29.7	0.012
No	8.1	37.8	

Hypertension plus low appendicular muscle mass			
Yes	19.2	30.8	0.650
No	11.5	38.5	

Hypertension plus low absolute muscle mass			
Yes	21.1	40.3	0.847
No	12.3	26.3	

Hypertension plus low sit-to-stand			
Yes	22.9	29.8	0.091
No	10.5	36.8	

Hypertension plus low walking speed			
Yes	20,4	44.4	0.991
No	11.1	24.1	

Hypertension plus low Janssen			
Yes	28.0	28.0	0.030
No	4.0	40.0	

Hypertension plus low Baumgartner			
Yes	13.2	23.5	0.363
No	16.2	47.1	

Hypertension plus low short physical performance battery			
Yes	24.6	26.3	0.006
No	7.0	42.1	

Data are shown as mean ± SD; MMSE = Mini-Mental State Examination.

**Table 4:** Results from multiple linear regression to predict MMSE.

Dependent variable	Predictor variable	Unstandardized beta	Standardized beta	*P*	sr^*2*^
MMSE	Absolute muscle mass (kg)	0.045	0.078	0.729	0.02
	Appendicular skeletal muscle mass (kg)	−0.144	−0.133	0.577	−0.03
	Janssen index (kg/m^2^)	0.065	0.146	0.322	0.05
	Baumgartner index (kg/m^2^)	0.026	0.078	0.507	0.04
	Walking speed (m/s)	−0.317	−0.024	0.836	−0.01
	Short physical performance battery (points)	0.342	0.147	0.201	0.07
	Sit-to-stand (s)	0.008	0.043	0.703	0.02
	HTN	−2.255	−0.407	0.002	−0.18

MMSE = Mini-Mental State Examination; HTN = hypertension; sr^2^ = square of semipartial correlation; *R* = 0.528, *R* ^2^ = 0.279; adjusted *R* ^2^.

## Supplementary Materials

Supplementary Materials Evaluating PP levels and salivary NO bioavailability according to SPPB, sit-to-stand, and SMI in the hypertensive volunteers.
