# Supplementary material for: Hypertension, Sarcopenia, and Global Cognitive Function in Community-Dwelling Older Women: A Preliminary Study
Source: J Aging Res. 2018 Jul 2;2018:9758040. doi: 10.1155/2018/9758040 (PMC6051132; doi:10.1155/2018/9758040)
Supplement: Supplementary Materials — Evaluating PP levels and salivary NO bioavailability according to SPPB, sit-to-stand, and SMI in the hypertensive volunteers. [file 9758040.f1.pdf]

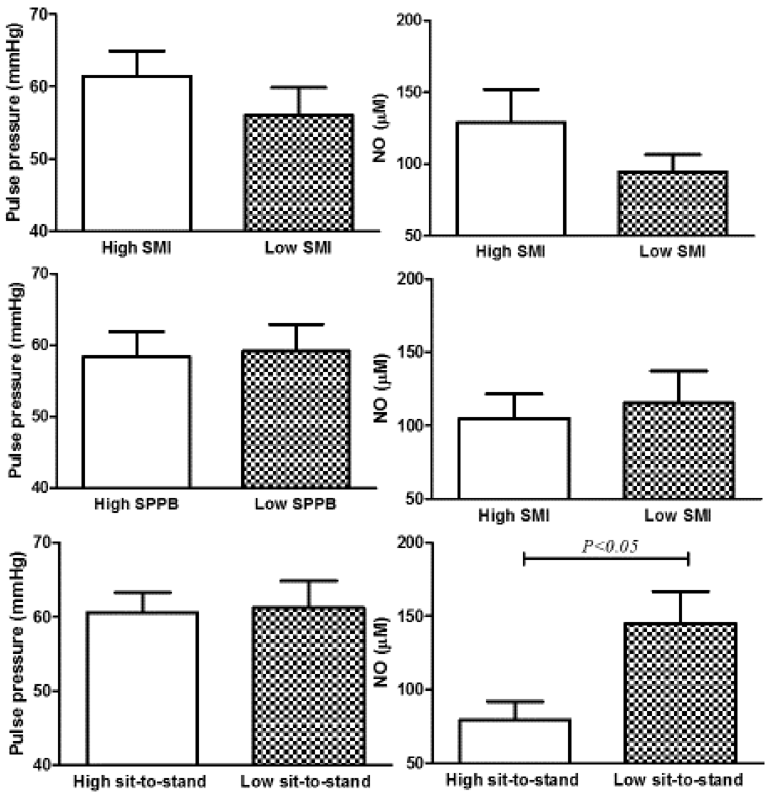

Figure 1 - SM. Pulse pressure and Nitric oxide (NO) bioavailability according to sarcopenic parameters in hypertensive patients.
